# Skeletal Muscle Cells Express ICAM-1 after Muscle Overload and ICAM-1 Contributes to the Ensuing Hypertrophic Response

**DOI:** 10.1371/journal.pone.0058486

**Published:** 2013-03-11

**Authors:** Christopher L. Dearth, Qingnian Goh, Joseph S. Marino, Peter A. Cicinelli, Maria J. Torres-Palsa, Philippe Pierre, Randall G. Worth, Francis X. Pizza

**Affiliations:** 1 Department of Kinesiology, The University of Toledo, Toledo, Ohio, United States of America; 2 Centre d'Immunologie de Marseille-Luminy U2M, Aix-Marseille Université, Marseille, France; 3 INSERM U631, Institut National de la Santé et Recherche Médicale, Marseille, France; 4 CNRS UMR6102, Centre National de la Recherche Scientifique, Marseille, France; 5 College of Medicine and Life Sciences, The University of Toledo, Toledo, Ohio, United States of America; University of Minnesota Medical School, United States of America

## Abstract

We previously reported that leukocyte specific β2 integrins contribute to hypertrophy after muscle overload in mice. Because intercellular adhesion molecule-1 (ICAM-1) is an important ligand for β2 integrins, we examined ICAM-1 expression by murine skeletal muscle cells after muscle overload and its contribution to the ensuing hypertrophic response. Myofibers in control muscles of wild type mice and cultures of skeletal muscle cells (primary and C2C12) did not express ICAM-1. Overload of wild type plantaris muscles caused myofibers and satellite cells/myoblasts to express ICAM-1. Increased expression of ICAM-1 after muscle overload occurred via a β2 integrin independent mechanism as indicated by similar gene and protein expression of ICAM-1 between wild type and β2 integrin deficient (CD18-/-) mice. ICAM-1 contributed to muscle hypertrophy as demonstrated by greater (p<0.05) overload-induced elevations in muscle protein synthesis, mass, total protein, and myofiber size in wild type compared to ICAM-1-/- mice. Furthermore, expression of ICAM-1 altered (p<0.05) the temporal pattern of Pax7 expression, a marker of satellite cells/myoblasts, and regenerating myofiber formation in overloaded muscles. In conclusion, ICAM-1 expression by myofibers and satellite cells/myoblasts after muscle overload could serve as a mechanism by which ICAM-1 promotes hypertrophy by providing a means for cell-to-cell communication with β2 integrin expressing myeloid cells.

## Introduction

The interplay between myeloid cells (neutrophils and macrophages) and skeletal muscle cells influences how the affected muscle responds and adapts to mechanical loading. The interplay is initiated by skeletal muscle cells which release factors that promote myeloid cell chemotaxis [1], [2], [3] resulting in their accumulation within skeletal muscle in the hours to days after mechanical loading and/or injury [4], [5], [6], [7], [8].

Myeloid cells are known for their role in muscle injury and subsequent repair/regeneration. We and others have reported that neutrophils injure cultured skeletal muscle cells [9], [10] and cause histological and/or functional abnormalities after contraction-induced muscle injury [5], [11]. Macrophages appear to produce both deleterious and beneficial outcomes in injured skeletal muscle. Specifically, macrophages have been reported to injure cultured skeletal muscle cells [10], [12], promote muscle repair/regeneration [7], [8], and enhance the migration, proliferation, and viability of satellite cells/myoblasts [3], [13], [14], which are required for muscle regeneration [15].

Myeloid cells also accumulate in skeletal muscle after non-injurious mechanical loading. Specifically, we have demonstrated that non-injurious protocols such as passive stretching, isometric contractions, and concentric contractions elevate myeloid cell numbers in skeletal muscle [4], [6] and promote myeloid cell chemotaxis in vitro [1]. Myeloid cell accumulation in non-injured skeletal muscle contributes to mechanical loading-induced adaptations, such as protection from subsequent injury [16] and hypertrophy [17], [18]. The mechanisms for how myeloid cells contribute to muscle plasticity after mechanical loading remain to be determined.

Effector functions of myeloid cells are initiated when they adhere to membrane structures of cells and/or to proteins of the extracellular matrix. This adhesion is facilitated by β2 integrins, which are leukocyte-specific heterodimeric glycoproteins composed of a common β subunit (CD18) and one of four α subunits (CD11a, CD11b, CD11c, or CD11d) [19], [20]. β2 integrins function as adhesion and signal transducing molecules that are important for neutrophil diapedesis, and the production of reactive oxygen species (ROS) and cytokines from myeloid cells [19], [20]. Using mice deficient in CD18 (CD18-/-), we found that β2 integrins contribute to skeletal muscle hypertrophy and influence the temporal pattern of satellite cell/myoblast proliferation and muscle differentiation after muscle overload [17]. We speculate that β2 integrins regulate the hypertrophic response to muscle overload by serving as a means by which myeloid cells bind to and initiate cell-to-cell communication with skeletal muscle cells.

A major ligand for the β2 integrin CD11b, which is primarily expressed by myeloid cells, is intercellular adhesion molecule-1 (ICAM-1; CD54) [20]. ICAM-1, a member of the immunoglobulin superfamily of adhesion molecules, consists of five extracellular domains, a transmembrane segment, and a cytoplasmic tail [20]. Vascular endothelial cells and leukocytes constitutively express ICAM-1 at low levels, and many different cell types express ICAM-1 in response to cytokines and ROS [20], [21], [22]. Induced expression of ICAM-1 by skeletal muscle cells has been reported after treatment of cultured human skeletal muscle cells with cytokines [23], [24], [25], [26] and in patients with inflammatory myopathies [27], [28]. No evidence however, exists on whether skeletal muscle cells in vivo express ICAM-1 under non-pathological conditions, such as after mechanical loading.

Membrane associated ICAM-1 functions as a point of attachment for leukocytes and as a signal transducer. The interaction of CD11b expressed by myeloid cells with ICAM-1 promotes their adhesion and production of ROS and cytokines [19], [20], [22]. Intracellular events in ICAM-1+ cells such as, activation of signaling pathways (e.g., MAPK and PI3K/Akt) [21], [22], [29] and protein synthesis of cytokines [30], [31], [32], are also initiated upon ligand binding to ICAM-1. Responses initiated by CD11b-ICAM-1 interactions are relevant to the hypertrophic response to muscle overload because MAPK, PI3K/Akt, and cytokines are known to regulate skeletal muscle cell proliferation, differentiation, and/or hypertrophy [33], [34], [35]. Thus, if skeletal muscle cells express ICAM-1 after muscle overload, then β2 integrin-ICAM-1 interactions could augment the hypertrophic response via adhesion-induced activation of myeloid cells, and/or via activation of ICAM-1 signaling in skeletal muscle cells.

Our initial objective was to test the hypothesis that muscle overload-induced hypertrophy of skeletal muscle is associated with increased gene and protein expression of ICAM-1 and its localization to myofibers and satellite cells/myoblasts. Because β2 integrins contribute to hypertrophy after muscle overload [17] and influence myeloid cell production of inducers of ICAM-1 expression (e.g., TNF-α and IL-1β) [19], [20], we also tested the hypothesis that β2 integrins promote ICAM-1 expression in skeletal muscle. This hypothesis was tested by comparing the expression profile of ICAM-1 in wild type and CD18-/- mice after muscle overload. Lastly, using wild type and ICAM-1-/- mice, we tested the hypothesis that ICAM-1 contributes to the hypertrophic response to muscle overload.

## Materials and Methods

### Animals

Male wild type (C57BL/6; Jackson Laboratory), CD18-/- (*Itgb2^tm1Bay^*; C57BL/6 background; Jackson Laboratory) and ICAM-1-/- (Icam1^tm1Cws^; C57BL/6 background; kindly provided by C. Wayne Smith, Baylor University) mice were tested at 3–4 months of age. The CD18-/- mice were hypomorphic homozygotes for the CD18 allele [36]; whereas, the ICAM-1-/- mice were generated by replacing the entire coding region of the ICAM-1 gene with a puromycin cassette [37]. Mice were housed on a 12 h light-dark cycle and fed standard laboratory chow and water ad libitum. Procedures were approved by the institutional animal care and use committee at the University of Toledo (Protocol number: 105399) and every effort was made to minimize animal suffering.

### Muscle Overload

Overload of plantaris muscles was achieved by bilateral removal of the gastrocnemius and soleus muscles as previously described [17]. Mice were permitted to ambulate for 3, 7, 14 or 21 days after surgical procedures. Mice who had normal cage activity and who did not undergo surgery were used as controls. Plantaris muscles were weighed and frozen for gene, protein, or histological analysis as previously described [17].

### Real Time PCR

RNA was isolated from plantaris muscles using a kit according to the manufacturer's protocol (Qiagen; Cat #: 74704). Purity and concentration of RNA was determined spectrophotometrically and reverse transcription was performed using the high capacity cDNA reverse transcription kit (Applied Biosystems). Real-time PCR was performed for ICAM-1 using TaqMan PCR master mix and gene expression primers and probes from Applied Biosystems (assay ID Mn00516023_m1). Each sample was run in triplicate and detection was achieved using Applied Biosystems 7500 Real-time PCR system. The magnitude of change in ICAM-1 relative to GAPDH (assay ID: Mn99999915_g1) was calculated using the relative quantification method (2^-ΔΔCT^).

### Western Blotting

Plantaris muscles and cultured skeletal muscle cells were homogenized in reducing sample buffer (2% sodium dodecyl sulphate, 1.5% dithiothreitol, 1M Tris-HCL and 10% glycerol) containing protease inhibitors (1 mM EDTA, 5μg/ml leupeptin, 5μg/ml aprotinin, and 11 mM 4-(2-aminoethyl) benzenesulfonyl fluoride) with a bead homogenizer (Tissue Lyser; Qiagen) and a cell sonicator (Misonix; Model S-4000), respectively. Samples were boiled and separated on 10% SDS-PAGE gels. Positive controls for ICAM-1 included lysates from a cell line of murine monocytes/macrophages (RAW 264.7 cells; Santa Cruz).

After electrophoresis, proteins were transferred to PDVF-FL (for ICAM-1) or nitrocellulose membranes (for Pax7) using a semi-dry protocol (20 V for 1 h in a solution containing 192 mM glycine, 25 mM Tris, and 10% methanol). Membranes were blocked with a Tris-buffered saline (TBS) solution containing 50% Odyssey® blocking buffer (for ICAM-1) or 1% casein (for Pax7). Membranes were washed in TBS/0.05% Tween-20 (TBS-T), cut at ∼40 or 75 kDa, and incubated overnight at 4 °C with an antibody for ICAM-1 (R&D Systems Cat# AF796; 1∶500), Pax7 (University of Iowa Developmental Studies Hybridoma Bank; 1∶200), GAPDH (Cell Signaling Cat# 2118; 1∶5,000), or α-tubulin (Cell Signaling Cat# 3873; 1∶750). GAPDH and α-tubulin served as a control for sample loading for whole muscle homogenates and cultured cell lysates, respectively. Membranes were washed and then incubated with an Alexa Fluor® 680 secondary antibody (1∶5,000–1∶10,000; Invitrogen). The relative abundance of ICAM-1, Pax7, GAPDH, and α-tubulin was quantified using the Odyssey® infrared detection system.

### Skeletal Muscle Cell Cultures

Expression of ICAM-1 was evaluated in C2C12 cells (ATCC) [38] and in primary skeletal muscle cells. Primary cultures were established by digesting hindlimb muscles of 5 d old wild type mice as described below and by a pre-plating method [39]. Pre-plating consisted of seeding cells digested from muscles into uncoated dishes for 2 h in isolation medium (Ham's F10 medium, 20% fetal bovine serum (FBS), 2.5 ng/ml of bFGF, 100 U/ml penicillin, 0.1 mg/ml streptomycin, and 0.25 ug/ml of amphotericin B; Sigma-Aldrich). Unattached cells were then plated in Matrigel® (BD Biosciences; Cat# 356234) coated dishes and allowed to proliferate in isolation medium. At ∼60% confluence, cells were collected and pre-plated for 1 h.

Relative purity of primary myoblasts was evaluated by morphology and by immunolabeling for Pax7, an established marker of satellite cells/myoblasts [15], [40]. Cytospin prepared slides of myoblasts were fixed in 4% formaldehyde, permeabilized with 0.5% triton X-100, blocked, and incubated with an anti-Pax7 antibody (University of Iowa Developmental Studies Hybridoma Bank; 1∶50). After incubation with a Cy3 conjugated secondary antibody, cells were mounted with Fluoromount-G containing 4',6-Diamidino-2- phenylindole (DAPI). Image analysis revealed that greater than 90% of the cultured primary cells were Pax7+.

Cells were seeded (2,500 cells/cm^2^) in growth medium (C2C12: DMEM and 10% FBS; primary myoblasts: 1∶1 DMEM/Ham's F10, 20% FBS, and 2.5 ng/ml of bFGF) and allowed to proliferate. C2C12 cells were seeded in non-coated wells; whereas, Matrigel® coated wells were used for primary cells. For differentiating cultures, C2C12 and primary cells were treated with differentiation medium (DMEM and 2% horse serum) for up to 6 d to induce the formation and maturation of multi-nucleated myotubes. Growth and differentiation media contained antibiotic-antimycotic reagents (100 U/ml penicillin, 0.1 mg/ml streptomycin, and 0.25 ug/ml of amphotericin B; Sigma-Aldrich) and were changed every 2 d.

To induce ICAM-1 expression, cultures containing proliferating myoblasts or differentiated myotubes (4 d in differentiation medium) were treated with 10 ng/ml of rmTNF-α (R&D Systems) for 24 h. ICAM-1 expression in proliferating cultures was assessed via flow cytometry and western blot; whereas, western blotting and immunofluorescence was used to detect ICAM-1 in differentiating cultures.

### Immunohistochemistry

Acetone fixed transverse sections (10 μm) were prepared for labeling [17] and treated with a primary antibody that recognizes an extracellular domain of mouse ICAM-1 (R&D Systems product # AF796; 1∶20), neutrophils (Ly6G antibody clone RB6-8C5; BD Pharmingen; 1∶100), or macrophages (F4/80 antibody clone CI:A3-1; Serotec Inc.; 1∶100). Slides serving as negative controls received PBS in place of the primary antibody. After incubation with a biotinylated anti-goat secondary antibody (1∶200) and then horseradish peroxidase (1∶1000), sections were developed with 3-amino-9-ethylcarbazole.

ICAM-1+ myofibers in one entire section of a muscle were manually counted and results were expressed as a percentage of the total number of myofibers within the section. The cross sectional area of ICAM-1+ and ICAM-1^neg^ myofibers were quantified using image analysis software (Image Pro Plus). The number and size of ICAM-1+ myofibers were determined only if myofiber membrane expression of ICAM-1 could be clearly delineated.

Ly6G+ and F4/80+ cells in two entire sections for each muscle were manually counted. The total number of cells were expressed relative to the volume of the section area (Ly6G+ or F4/80+/mm^3^) [17].

### Confocal Microscopy

Transverse sections (25 μm) of muscles were fixed in 50% acetone/50% methanol, blocked, and incubated with an ICAM-1 antibody (R&D Systems product # AF796; 1∶20), and an antibody that recognizes the CD31 (endothelial cell marker; BD Pharmingen Catalog # 550274; 1∶50) or CD11b antigen (myeloid cell marker; clone M1/70; BD Pharmingen product # 550282; 1∶50). Myofibers were delineated using fluorescent wheat germ agglutinin (WGA; Alexa Fluor® 633; 1∶500) [41], which binds to sialic acid and N-acetylglucosaminyl residues of glycoproteins that are on the sarcolemma of myofibers, and links the sarcolemma to the basal lamina [42], [43]. Prior work has also demonstrated that WGA co-localizes with both laminin and dystrophin [42], [43], which are commonly used markers of the basal lamina and sarcolemma of myofibers, respectively. Because of the ubiquitous expression of glycoproteins, WGA will also delineate other cells residing in skeletal muscle, as well as connective tissue. Sections were mounted with Fluoromount-G containing DAPI and analyzed using a TCS SP5 multi-photon laser scanning confocal microscope (Leica Microsystems) at The University of Toledo's Advanced Microscopy and Imaging Center.

### Histology

Transverse sections (10μm) were stained with hematoxylin and eosin and examined for signs of injury, necrosis and regeneration. The number of injured, necrotic, and central nucleated (regenerating) myofibers was determined as previously described [17]. Normal myofibers were defined as those that did not show signs of injury, necrosis, or regeneration. The total number of myofibers in hematoxylin and eosin stained sections of overloaded muscles was manually counted and injured, necrotic, and regenerating myofibers were expressed as a percentage of the total number of myofibers within a muscle section.

We also performed immunolabeling for mouse IgG (1∶200 of anti-mouse IgG-Cy3; Jackson ImmunoResearch Laboratories) in acetone fixed sections to reveal discontinuities in the membrane of myofibers. The presence of IgG in the cytoplasm of myofibers, suggestive of membrane lesions, was detected via fluorescent microscopy and used as an additional measure of myofiber injury. Double labeling was performed to determine if ICAM-1 was expressed on the membrane of injured and/or regenerating myofibers.

### Muscle Digestion

Plantaris muscles from a single mouse were pooled, minced in DMEM, centrifuged, and then incubated at 37 °C in DMEM containing 0.1% pronase (Calbiochem) for 1 h. Two plantaris muscles from a single mouse represent a sample size of one. Cell suspensions were filtered (60 μm; Millipore), centrifuged, and suspended in staining buffer. The number of cells isolated was determined using a hemacytometer.

### Flow Cytometry

Flow cytometry was performed on cells isolated from digested plantaris muscles and in cultured C2C12 and primary myoblasts. Cultured cells were collected from tissue culture dishes using StemPro® Accutase® according to the manufacturer's protocol (Invitrogen).

Cells were treated (30 min at 4 °C) with Fc block (BD Biosciences) and 200–500,000 cells were incubated for 30 min with one or more fluorophore-conjugated antibodies to identify leukocytes (0.5 ug of FITC-CD45; BD Biosciences), ICAM-1 (0.4 ug of PE-CD54 clone YN1/7.4; eBiosciences), satellite cells/myoblasts (0.5 ug of Alexa Flour® 649-integrinα7; Fabio Rossi, University of British Columbia), or endothelial cells (0.5 ug of FITC-CD31; BD Biosciences). Isotype control antibodies were used in antibody titration experiments to control for non-specific binding. Cells were analyzed using the BD Biosciences FACSAria (for digested muscle) or FACSCalibur (for cultured cells) at the University of Toledo Flow Cytometry core facility.

To increase the relative detection of satellite cells/myoblasts (integrin α7+ cells) [44] in cell suspensions of digested muscles, flow cytometry gates were set on viable cells using propidium iodide, non-leukocytes (CD45^neg^) and non-endothelial cells (CD31^neg^). Thus, satellite cells/myoblasts were operationally defined as viable cells that were CD45^neg^CD31^neg^integrin α7+ [45]. The number of ICAM-1+ satellite cells/myoblasts for each sample was calculated by multiplying the total number of isolated cells by the fraction of the total number of cells that were CD45^neg^CD31^neg^ integrin α7+ICAM-1+. This number was divided by the combined mass of plantaris muscles from a single mouse to control for overload-induced changes in muscle mass.

### Markers of Hypertrophy

Wet mass of plantaris muscles, total protein content, and the size of myofibers served as outcomes measures of hypertrophy. Total protein content of control and overloaded (14 and 21 d) plantaris muscles was quantified as previously described [17]. The cross-sectional area of normal myofibers in hematoxylin and eosin stained sections of control and 14 d overloaded plantaris muscles were quantified using image analysis software as previously described [17]. The cross-section area of normal myofibers was determined only if the membrane could be clearly delineated by the image analysis software (Image Pro Plus).

### Protein Synthesis

Protein synthesis was measured in control and 7 d overloaded muscles using a nonradioactive technique known as surface sensing of translation (SUnSET) [46], [47]. Plantaris muscles were suspended in 2.0 ml eppendorf tubes by tying a suture to the distal tendon of each muscle and securing the suture to the cap of a tube. Muscles were then bathed in 1.0 ml of DMEM containing 1 uM puromycin (an analog of tyrosyl-tRNA) for 30 min at 37 °C. The viability of muscles was maintained by constant bubbling of a high oxygen gas mixture (95% O_2_/5% CO_2_) through caps of eppendorf tubes.

For western blot detection of puromycin, homogenates of plantaris muscles were boiled, separated on 10% SDS-PAGE gels (50 ug/lane), and then transferred to PDVF-FL using a wet transfer protocol (200 mA for 1 h in 192 mM glycine, 25 mM Tris, and 10% methanol). Membranes were blocked in 5% non-fat dry milk, washed with TBS-T and incubated overnight at 4 °C with mouse anti-puromycin (1∶5,000 in TBS-T/5% BSA) [46], [47]. Membranes were washed in TBS-T and incubated with an isotype specific secondary antibody (Alexa Fluor® 680; 1∶10,000 in TBS-T/5% BSA). The incorporation of puromycin into proteins was quantified by measuring the density of the entire lane [47] using the Odyssey® infrared detection system.

### Statistical Analyses

Data sets were analyzed with either a one-way or a two-way analysis of variance (ANOVA) using SigmaStat. The Student Newman-Keuls post-hoc test was used to locate the differences between means when the observed F ratio was statistically significant (p<0.05). Data are reported as mean and standard error.

## Results

### Increased Expression of ICAM-1 in Overloaded Muscles

**Figure 1:**
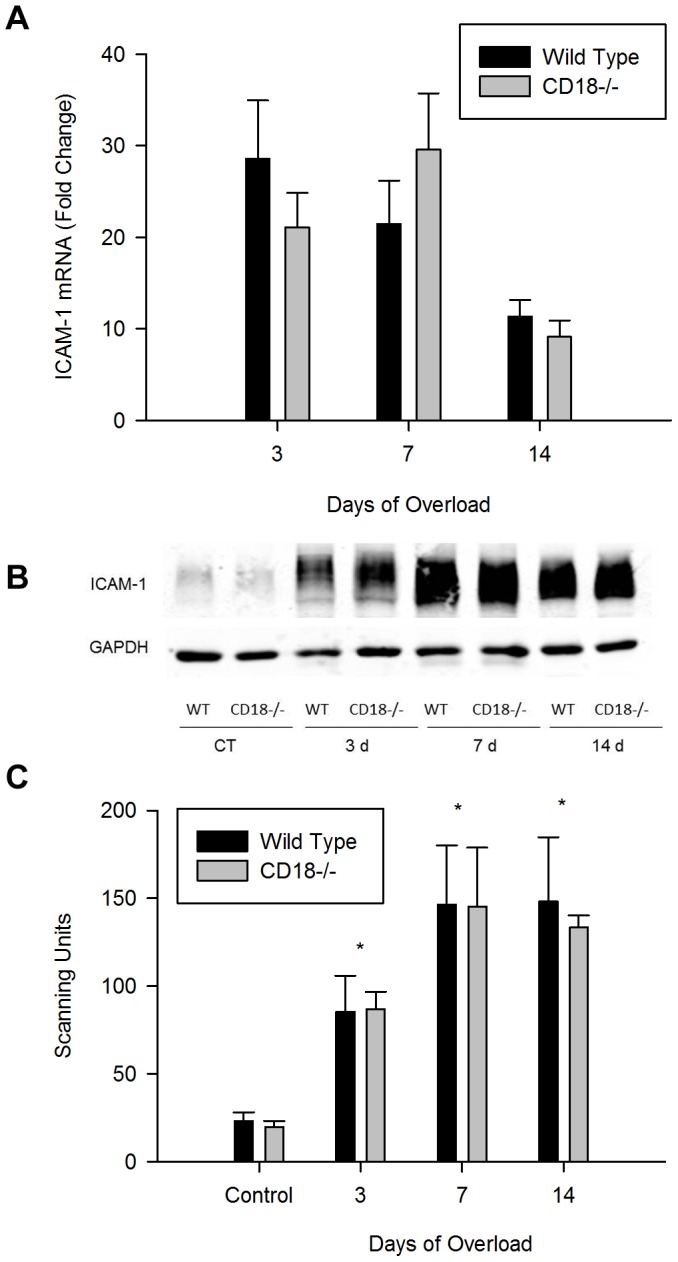
Gene and protein expression of ICAM-1. A) Fold change in ICAM-1 gene expression (n = 8/group). Gene transcripts tended (p = 0.054) to be lower at 14 d relative to 3 d of overload for both strains of mice. B) Representative western blot of ICAM-1 (110 kDa) in control and overloaded (3, 7, and 14 d) muscles of wild type and CD18-/- mice (15 ug of protein/lane). C) Quantitative analysis of ICAM-1 protein (n = 7/group). *, significantly elevated at each overload time point compared to control levels for both strains of mice.

For wild type mice, muscle overload increased gene expression of ICAM-1 by 10–30 fold and the relative abundance of ICAM-1 protein by 4–6 fold (Figure 1). In overloaded CD18-/- mice, gene and protein expression of ICAM-1 were elevated to levels that were similar to those found for overloaded wild type mice. These findings indicate that β2 integrins do not influence the expression profile of ICAM-1 in skeletal muscle after muscle overload.

### Protein Expression of ICAM-1 in Cultured Skeletal Muscle Cells

**Figure 2:**
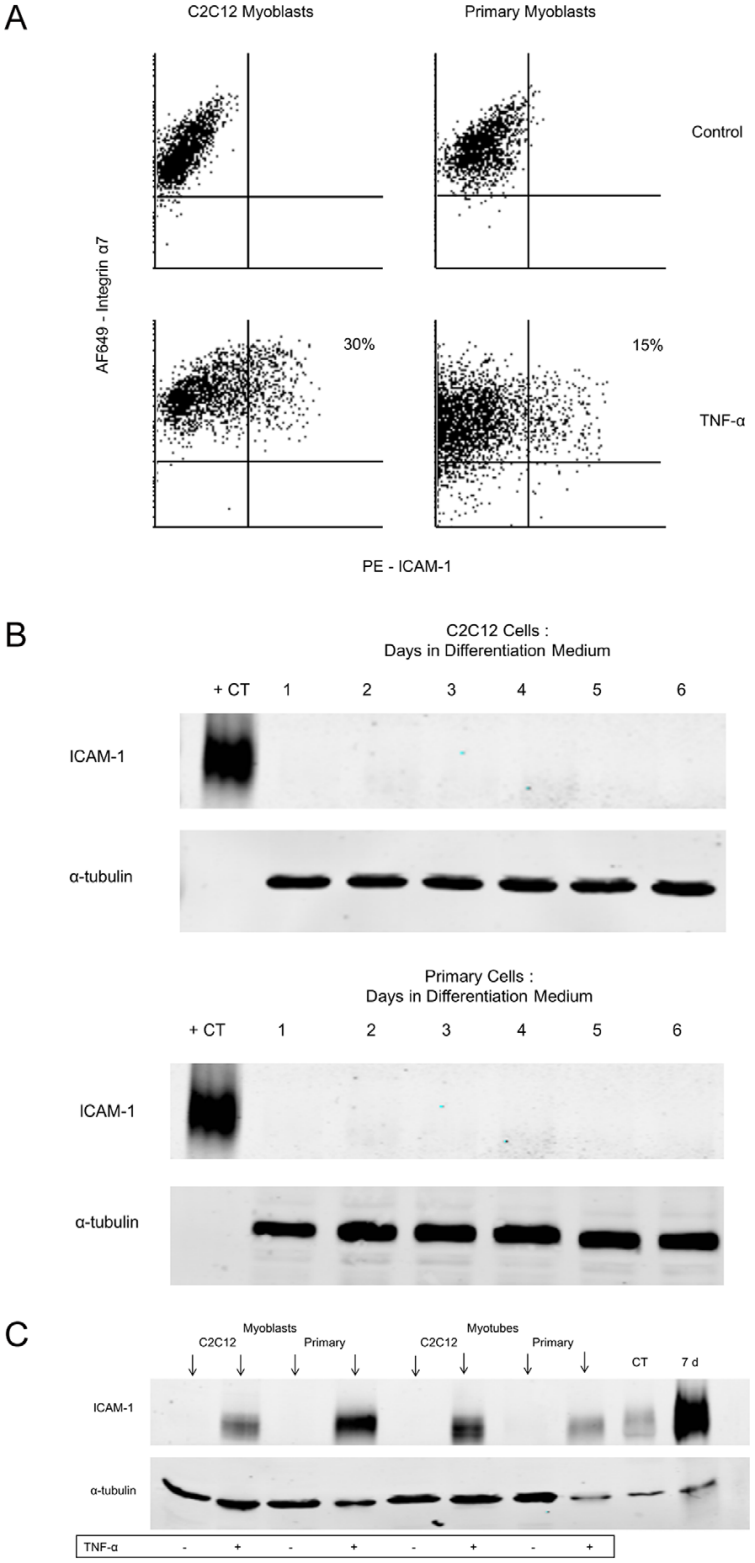
ICAM-1 expression by cultured skeletal muscle cells. A) Representative flow cytometric quadrant plots of control and TNF-α treated (10 ng/ml for 24 h) cultures of myoblasts. ICAM-1 was detected using a phycoerythrin (PE) conjugated antibody; whereas, myoblasts were identified using an AlexaFlour® 649 (AF649) α7 antibody. ICAM-1 was not expressed by C2C12 and primary myoblasts in control cultures; whereas, TNF-α treatment caused ICAM-1 to be expressed by 30% and 15% of C2C12 and primary myoblasts, respectively. B) Representative western blots for ICAM-1 expression in C212 and primary cells treated with differentiation medium for up to 6 d (20 ug of protein/lane). A cell lysate of a myeloid cell line (RAW 264.7 cells; 5 ug of protein) was used a positive control (+CT). Membranes were probed for α-tubulin (50 kDa) to serve as a control for sample loading. ICAM-1 was not detected in differentiating or differentiated cultures. C) Representative western blot for ICAM-1 after treating proliferating myoblasts or differentiated myotubes (4 d in differentiation medium) with TNF-α (10 ng/ml for 24 h) (20 ug of protein/lane). TNF-α treatment resulted in a ICAM-1 band that was of the same molecule weight (110 kDa) as those found in plantaris muscles from control (CT) and 7 d overloaded (7 d) wild type mice (15 ug of protein/lane).

Control cultures of proliferating, differentiating, or terminally differentiated skeletal muscle cells (C2C12 and primary) did not express ICAM-1 (Figure 2). Flow cytometry revealed that TNF-α treatment induced ICAM-1 expression in 30% and 15% of C2C12 and primary myoblasts, respectively. In differentiated cultures, multinucleated myotubes and non-fused myoblasts were found to express ICAM-1 after TNF-α treatment (Figure S1). Proliferating and differentiated cultures (C2C12 and primary cells) treated with TNF-α showed a prominent ICAM-1 band appearing at 110 kDa. This band was of the same molecular weight as the ICAM-1 band found in homogenates of control and overloaded plantaris muscles. Because several cell types in overloaded muscles were found to express ICAM-1, we interpret these findings to indicate that the increased ICAM-1 protein expression seen in homogenates of overloaded muscles includes ICAM-1 expressed by skeletal muscle cells.

### Endothelial Cell (CD31+) Expression of ICAM-1 in Skeletal Muscle

**Figure 3:**
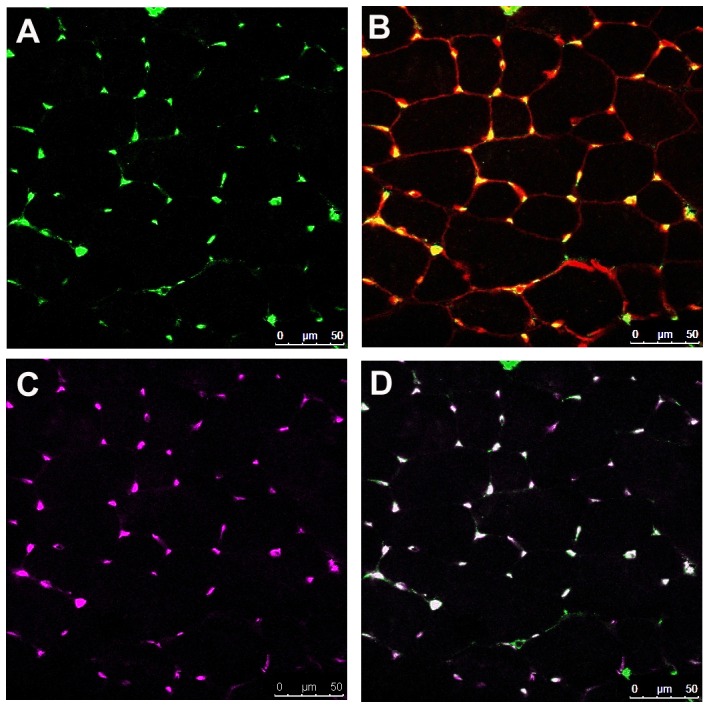
ICAM-1 localization in control muscle of wild type mice. Representative images from confocal microscopy. A) ICAM-1 (green), B) WGA (membrane marker; red) and ICAM-1 (green), C) CD31 (endothelial cell marker; purple), and D) Merged image of ICAM-1 (panel A), and CD31 (panel C). Co-localization analysis revealed that the majority (90–95%) of the ICAM-1 labeling in control muscles was expressed by CD31+ endothelial cells. ICAM-1 was not found on the membrane of myofibers in control muscles.

Co-localization analysis revealed that 90–95% of the ICAM-1 found in sections of control muscles was localized to CD31+ endothelial cells (Figure 3). Blood vessels in control muscles were also found to express ICAM-1 (Figure S2). These findings are consistent with the constitutive expression of ICAM-1 by endothelial cells in the vasculature of skeletal muscle [48]. Importantly, ICAM-1 was not found on the membrane of myofibers in control muscles.

**Figure 4:**
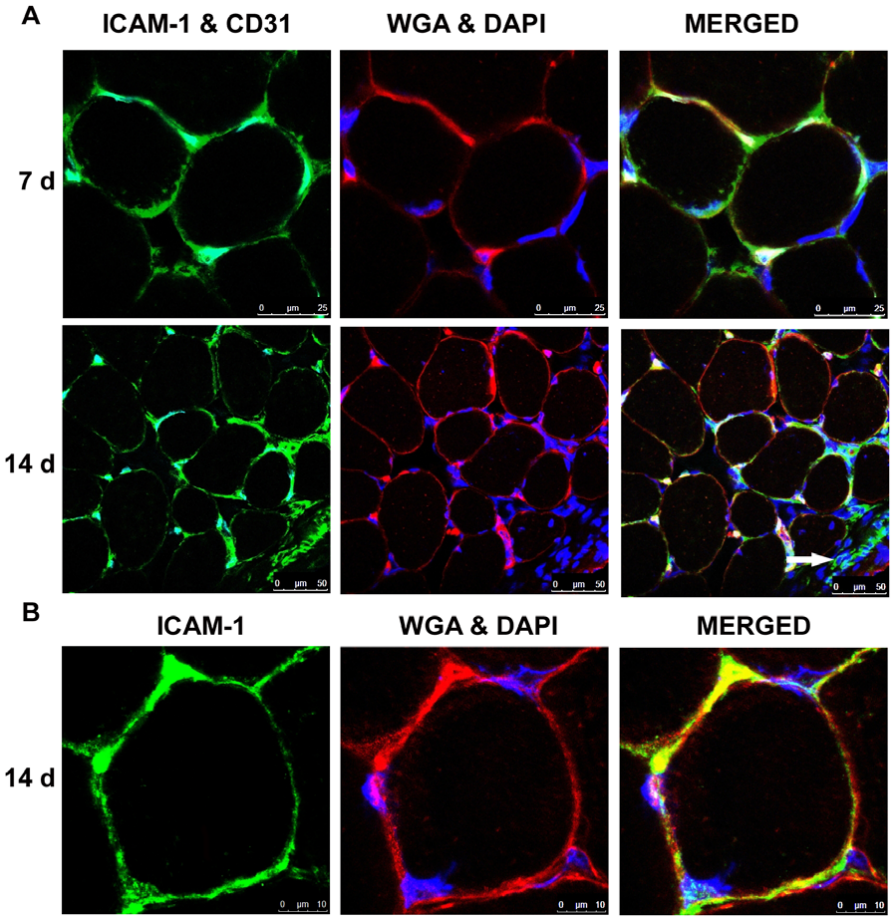
ICAM-1 localization in 7 and 14 d overloaded muscles of wild type mice. Representative images from confocal microscopy. A) ICAM-1 (green) was found to be co-localized to CD31+ endothelial cells (cyan) and to be associated with the membrane of myofibers (WGA+; red). Cells (DAPI+; blue) in the interstitium were also found to express ICAM-1 (arrow). Column labeled as “MERGED” represents an overlay of the ICAM-1, CD31, WGA and DAPI images. B) Higher magnification clearly revealed the colocalization of ICAM-1 (green) with the membrane marker WGA (red) in overloaded muscles. Column labeled as “MERGED” include images of ICAM-1, WGA, and DAPI.

In overloaded muscles (Figure 4), the number of CD31+ ICAM-1+ cells in a muscle section was similar to that observed in control muscles. However, the intensity of ICAM-1 labeling in CD31+ cells in overloaded muscles was noticeably greater than that observed in control muscles prior to image capture. This resulted in the use of lower settings for image capture of ICAM-1 in double labeled sections of overloaded compared to control muscles. This difference may represent increased endothelial cell expression of ICAM-1 after muscle overload.

### Myeloid Cell (CD11b+) and Leukocyte (CD45+) Expression of ICAM-1 in Overloaded Muscles

**Figure 5:**
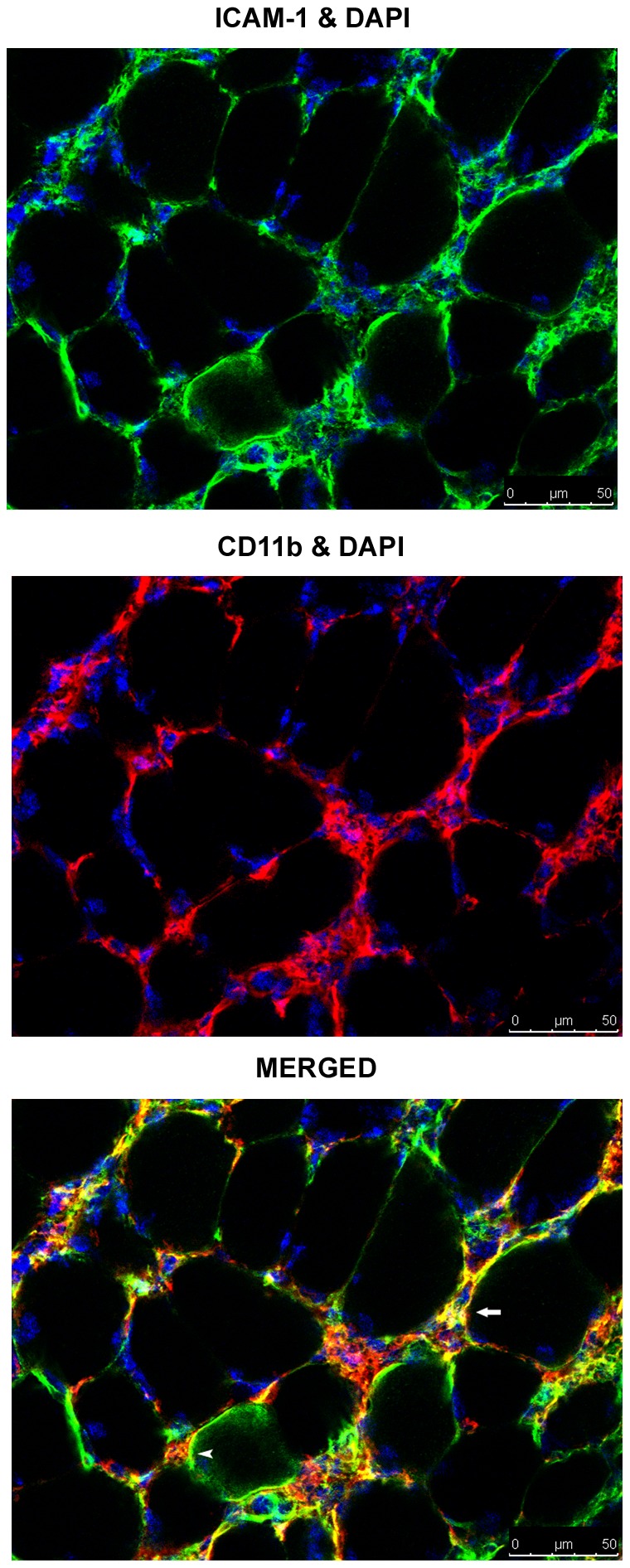
ICAM-1 and CD11b localization in 7 d overloaded muscle of wild type mice. Representative images from confocal microscopy. ICAM-1 (green) and CD11b (red) co-localized on the membrane of myofibers (arrow) and CD11b+ cells were closely associated with ICAM-1+ myofibers (arrowhead). Numerous CD11b+ cells residing in overloaded muscles expressed ICAM-1.

Cells residing in the interstitium of overloaded muscles were also found to express ICAM-1 (Figure S2) and to be CD31^neg^ (Figure 4) and CD11b+ (Figure 5). Flow cytometry revealed that 84% of the CD45+ cells found in 7 d overloaded muscles (n = 4) of wild type mice expressed ICAM-1; whereas, 52% of the CD45+ cells in control muscles (n = 3) were ICAM-1+. These data demonstrate myeloid cell expression of ICAM-1 and increased expression of ICAM-1 by leukocytes in overloaded muscles.

### Myofiber Expression of ICAM-1 in Overloaded Muscles

In contrast to myofibers in control muscles, immunohistochemical (Figure S2) and immunofluorescent labeling revealed ICAM-1 on the membrane of myofibers in overloaded muscles of wild type (Figures 4 and 5) and CD18-/- (Figure S3) mice. Myeloid cells (CD11b+) were found to be closely associated with ICAM-1+ myofibers (Figure 5). Although very few injured or necrotic myofibers were found in overloaded muscles (<0.5% of the total number of myofibers), ICAM-1 was not on the membrane of injured or necrotic myofibers. Few regenerating myofibers, which are prevalent at 7 d of overload [17], expressed ICAM-1 (∼1.0% of the total number of regenerating myofibers were ICAM-1+).

The percentage of ICAM-1+ myofibers was 9, 18, and 20% at 3, 7, and 14 d of overload, respectively. At 14 d of overload, the mean cross sectional area of ICAM-1+ myofibers was 20% larger (2441±195 μm^2^) than myofibers that did not express ICAM-1 (1958±87 μm^2^). We interpret this finding to indicate that myofiber expression of ICAM-1 is fundamentally important to the hypertrophic response.

### Satellite cells/Myoblasts Expression of ICAM-1 in Overloaded Muscles

**Figure 6:**
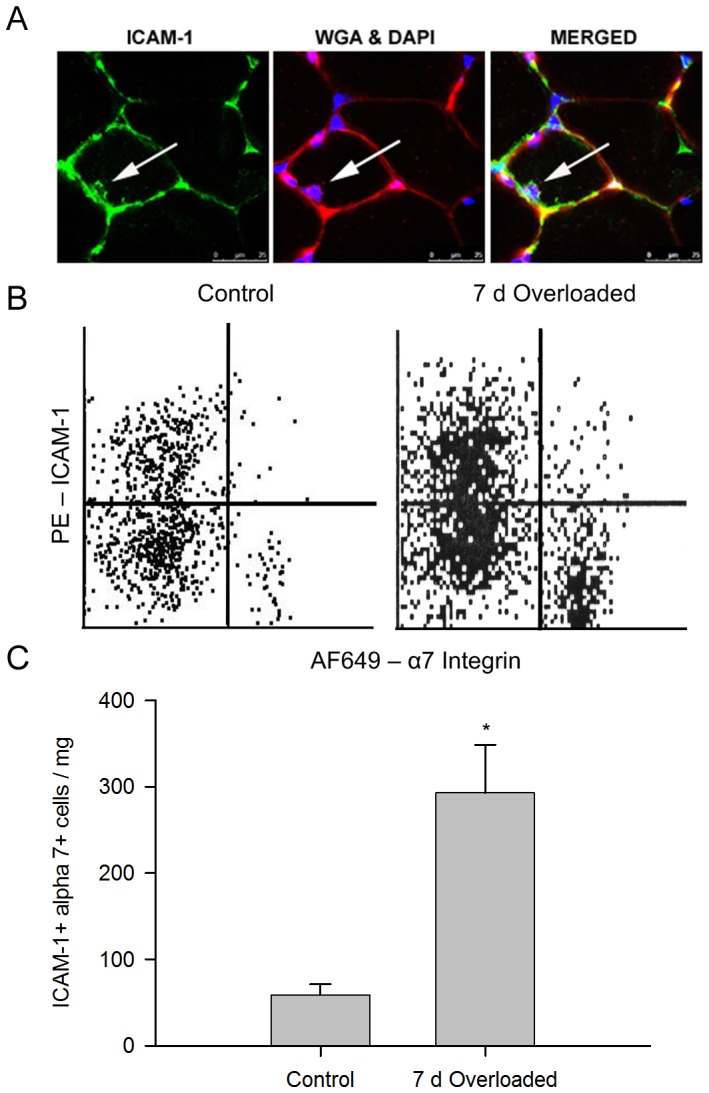
ICAM-1 expression by satellite cells/myoblasts. A) Confocal images of wild type muscle overloaded for 7 d. Arrow indicates a presumptive satellite cell that is positive for ICAM-1. B) Representative plots from flow cytometric analysis of cells isolated from control and 7 d overloaded muscles of wild type mice. ICAM-1 was detected using a phycoerythrin (PE) conjugated antibody; whereas, satellite cells/myoblasts were identified using an AlexaFlour® 649 (AF649) α7 antibody. Satellite cells/myoblasts were operational defined as CD45^neg^CD31^neg^ integrin α7+cells. C) Flow cytometric determination of ICAM-1 expression by satellite cells/myoblasts (CD45^neg^CD31^neg^ integrin α7+ICAM-1+) expressed relative to muscle mass (mg). *, significantly higher for 7 d overloaded muscles (n = 6) compared to control muscles (n = 4).

Satellite cells, which normally reside in a niche between the sarcolemma and the basal lamina of myofibers [34], appeared to express ICAM-1 in overloaded muscles (Figure 6). Indeed, flow cytometry of cells isolated from plantaris muscles revealed that the number of ICAM-1+ satellite cells/myoblasts (CD45^neg^CD31^neg^integrin α7+) was 5 fold higher in overloaded muscles compared to control muscles. The percentage of the cells that were ICAM-1+ satellite cells/myoblasts was also higher in overloaded (2.1±0.4%) compared to control muscles (0.8±0.2%). These findings demonstrate that a population of satellite cells/myoblasts expresses ICAM-1 in hypertrophying muscles.

### ICAM-1 Contributes to Hypertrophy induced by Muscle Overload

**Figure 7:**
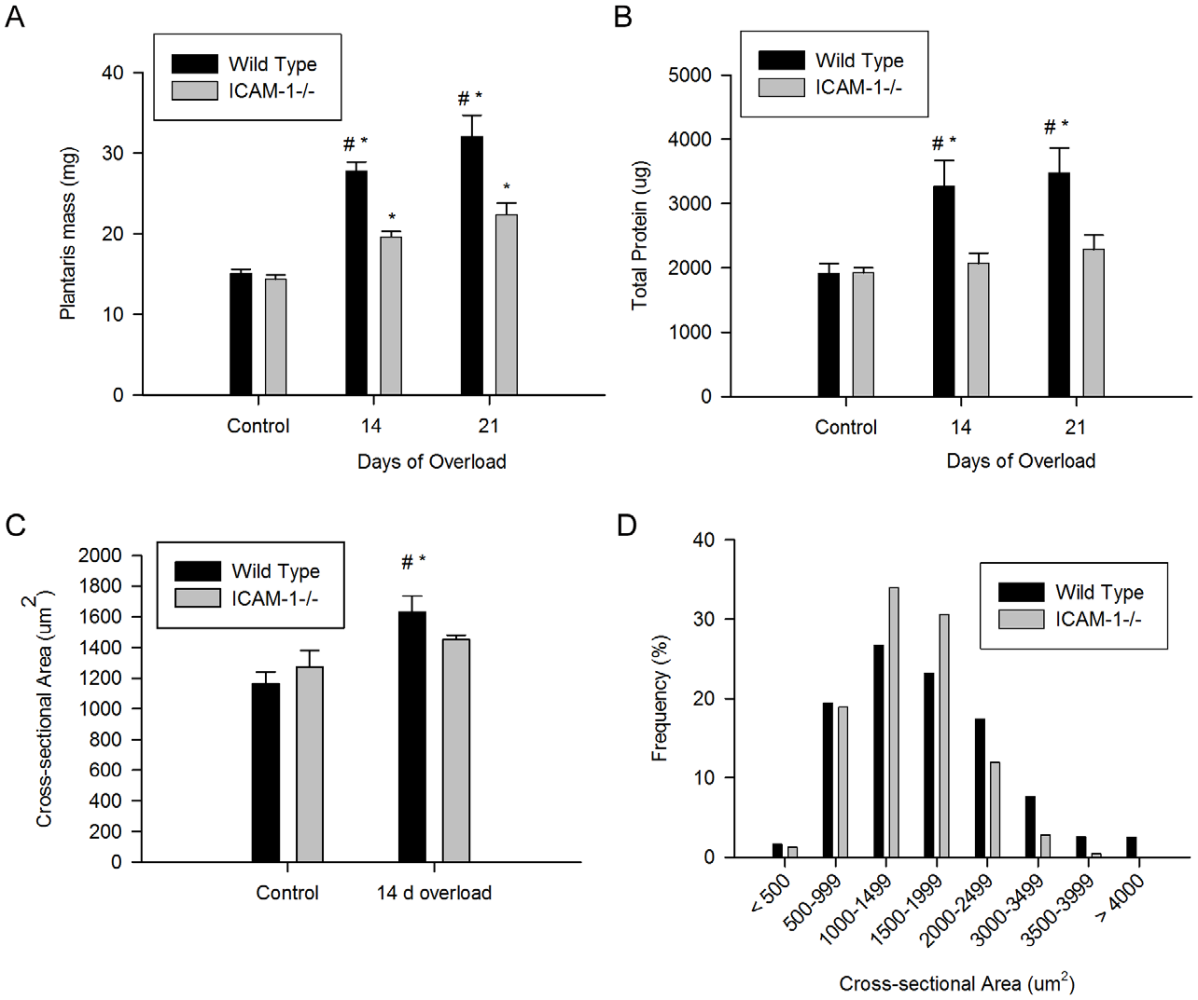
Measures of skeletal muscle hypertrophy. A) Wet plantaris mass in control and overloaded mice (n = 16-20/group). B) Total protein in muscle homogenates of control (n = 6/group) and overloaded mice (n = 8/group). C) Mean cross-sectional area of normal myofibers in control (n = 1813 and 1697 myofibers for wild type and ICAM-1-/- mice, respectively)/strain) and 14 d overloaded (n = 2617 and 1096 myofibers for wild type and ICAM-1-/- mice, respectively) muscles. D) Frequency distribution of the size of normal myofibers at 14 d of overload #, significant interaction at 14 and 21 d of overload. *, significantly higher at 14 and/or 21 d of overload relative to respective controls.

**Figure 8:**
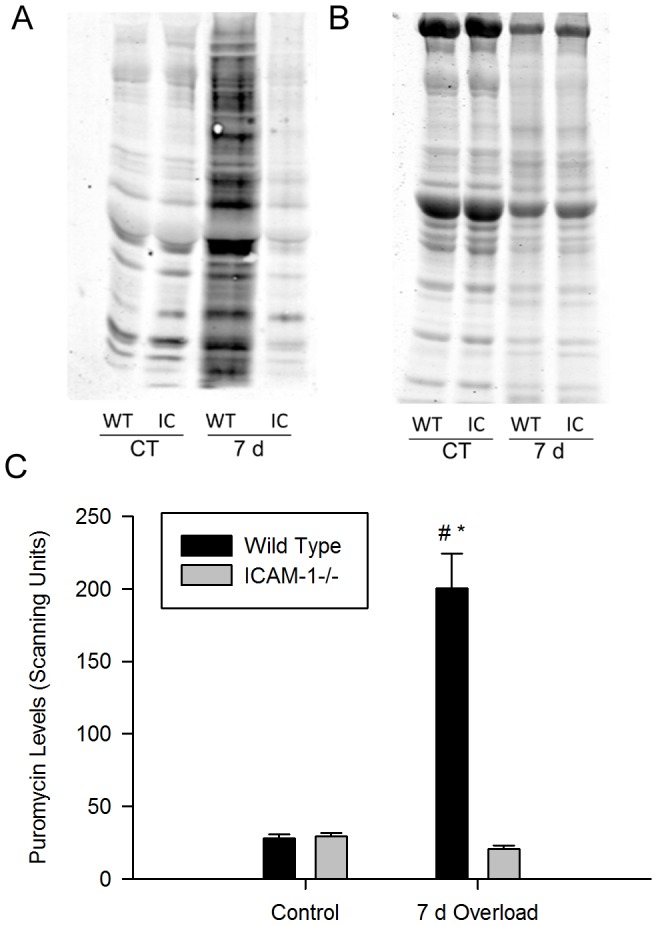
Protein synthesis, measured using the nonradioactive SUnSET technique, in plantaris muscles. A) Representative western blot (50 ug of protein/lane) of puromycin in control (CT) and 7 d overloaded muscles of wild type (WT) and ICAM-1-/- (IC) mice. B) Coomassie blue stained 10% SDS PAGE gel containing the same samples shown in panel A. C) Quantitative analysis of the relative abundance of puromycin incorporation into proteins (n = 7-8/group). #, significant interaction at 7 d of overload. *, significantly elevated at 7 d of overload compared to controls for wild type mice.

To test the contribution of ICAM-1 to hypertrophy, we exposed plantaris muscles of wild type and ICAM-1-/- mice to muscle overload. Body mass (data not reported), plantaris mass, total protein content, and myofiber size were similar in control muscles of wild type and ICAM-1-/- mice (Figure 7). Muscle overload increased muscle mass, muscle protein content, and myofiber size in wild type mice to levels that were higher than those found in ICAM-1-/- mice. The total number of myofibers in muscle sections of 14 and 21 d overloaded muscles however, was similar between wild type and ICAM-1-/- mice (data not reported). In overloaded wild type mice, muscle mass and protein content were elevated above control levels by ∼2 fold; whereas, myofiber size was ∼30% larger than control values. In overloaded ICAM-1-/- mice, muscle mass was elevated above control levels; whereas, total protein content and myofiber size remained at control levels. Differences in measures of hypertrophy between wild type and ICAM-1-/- mice were corroborated by measures of muscle protein synthesis. Specifically, using the nonradioactive SUnSET technique [46], [47] we found that protein synthesis in 7 d overloaded muscles was 10 fold greater for wild type compared to ICAM-1-/- mice (Figure 8). Protein synthesis remained at control levels in 7 d overloaded muscles of ICAM-1-/- mice. Thus, ICAM-1-/- mice showed little to no signs of hypertrophy after muscle overload.

### ICAM-1 influences Pax7 expression and Regenerating Myofibers after Muscle Overload

**Figure 9:**
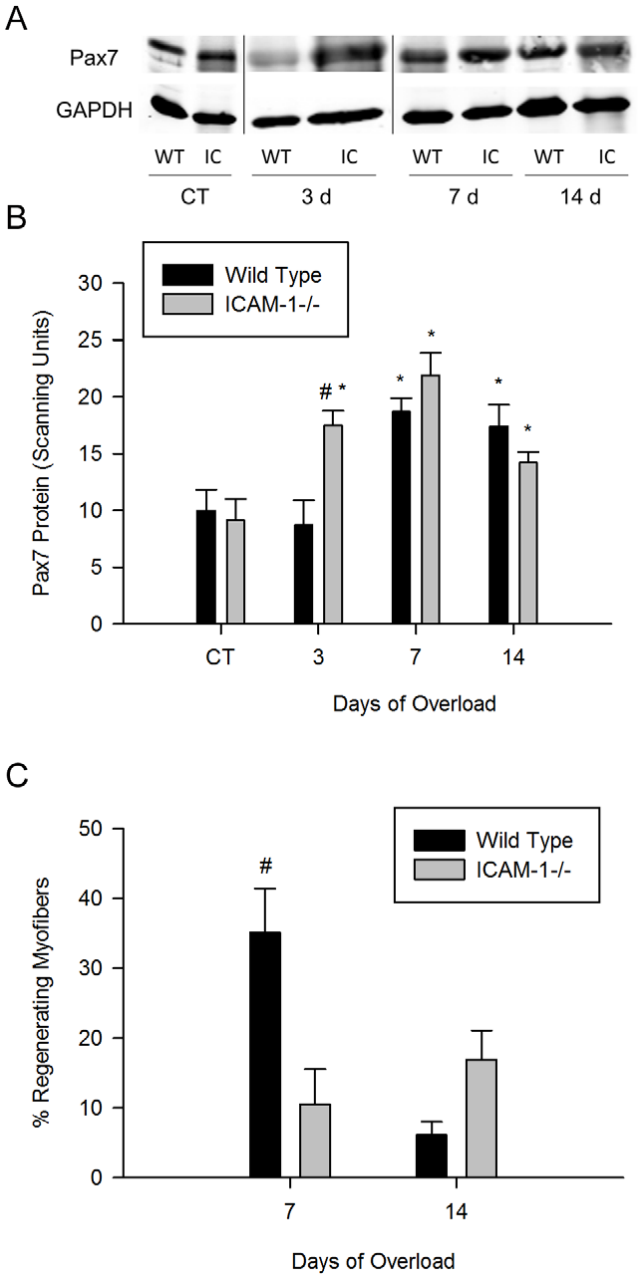
Satellite cells/myoblasts and regenerating myofibers after muscle overload. A) Representative western blot (30 ug of protein/lane) of Pax7 (60 kDa) in control (CT) and overloaded (3, 7 and 14 d) muscles of wild type (WT) and ICAM-1-/- (IC) mice. B) Quantitative analysis of the relative abundance of Pax7 protein (n = 6–8/group). #, significant interaction at 3 d of overload. *, significantly elevated at each overload time point compared to control levels for both wild type and ICAM-1-/- mice. C) Regenerating myofibers expressed as a percentage of the total number of myofibers. #, significant interaction at 7 d of overload.

At 3 d of overload, the relative abundance of Pax7, a marker of satellite cells/myoblasts [15], [40], remained at control levels for wild type mice and increased by ∼2 fold in ICAM-1-/- mice (Figure 9). However, at 7 and 14 d of overload, the relative abundance of Pax7 increased above control levels by a similar magnitude in wild type and ICAM-1-/- mice. Our findings indicate that the expression of ICAM-1 contributes to the acute regulation of the number of satellite cells/myoblasts in overloaded muscles.

The percentage of regenerating myofibers at 7 d of overload was 3.5 fold higher for wild type compared to ICAM-1-/- mice (Figure 9). At 14 d of overload, the percentage of regenerating myofibers were similar (p = 0.11) in wild type compared to ICAM-1-/- mice. These findings indicate that ICAM-1 augments the kinetics of regenerating myofiber formation and their subsequent maturation into normal myofibers with peripheral nuclei after muscle overload.

### ICAM-1 Contributes to Myeloid Cell Accumulation after Muscle Overload

**Figure 10:**
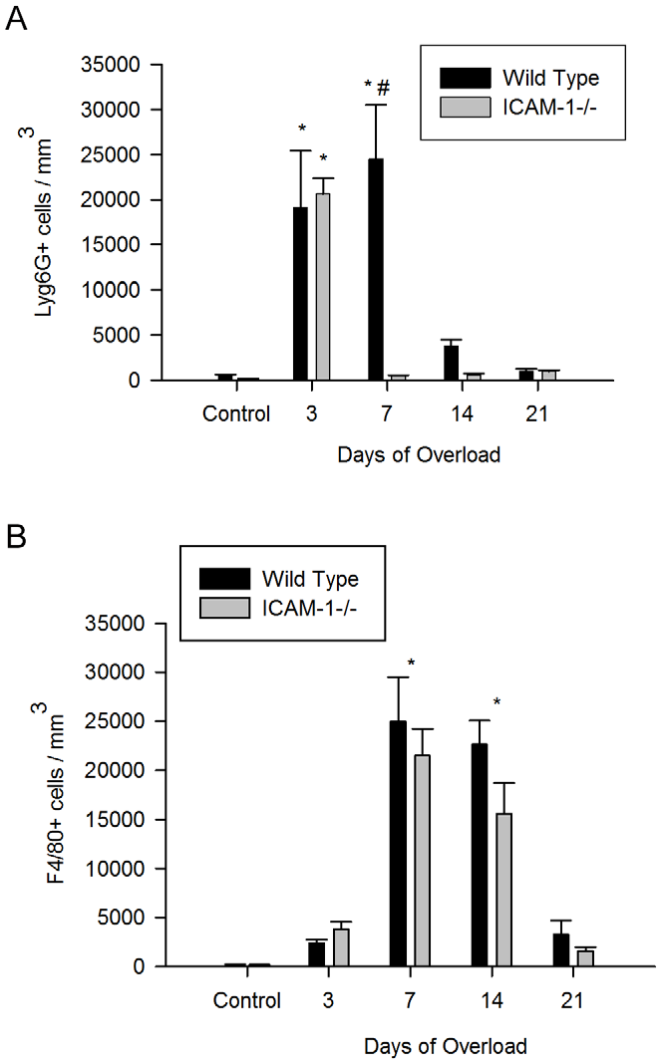
Myeloid cell accumulation in plantaris muscles. A) Neutrophil concentrations (Ly6G+ cells/mm^3^; n = 6–8/group). *, elevated relative to respective controls. #, higher for wild type compared to ICAM-1-/- mice at 7 d of overload. B) Macrophage concentrations (F4/80+ cells/mm^3^; n = 6-8/group). *, elevated relative to controls.

At 3 d of overload, neutrophil concentrations were elevated in both wild type and ICAM-1-/- mice relative to controls; whereas, macrophages remained at control levels for both wild type and ICAM-1-/- mice (Figure 10). At 7 d of overload, neutrophils were 51 fold higher in wild type mice compared to ICAM-1-/- mice; whereas, macrophage concentrations were similar between wild type mice and ICAM-1-/- mice. Neutrophils returned to control levels at 14 d of overload in wild type mice; whereas, macrophages were still elevated at 14 d of overload and returned to control levels by 21 d in both wild type and ICAM-1-/- mice. These findings demonstrate that ICAM-1 contributes to the accumulation profile of neutrophils in a time dependent manner.

## Discussion

The present study was conducted to explore the possibility that β2 integrins promote hypertrophy after muscle overload [17] via a mechanism that is dependent on ICAM-1 expression. A major finding of the present study was that muscle overload increased gene and protein expression of ICAM-1 in muscle homogenates and induced ICAM-1 expression on the membrane of myofibers and satellite cells/myoblasts (CD45^neg^CD31^neg^ integrin α7+). The similarity in the expression profile of ICAM-1 in wild type and CD18-/- mice after muscle overload demonstrated that increased expression of ICAM-1 occurred via a β2 integrin independent mechanism. Importantly, expression of ICAM-1 contributed to the hypertrophic response as indicated by greater overload-induced elevations in muscle protein synthesis, mass, protein content, and myofiber size in wild type compared to ICAM-1-/- mice. As ICAM-1 was found to be expressed by myofibers, satellite cells/myoblasts, endothelial cells, and leukocytes residing in overloaded muscles, we cannot determine the individual contribution of each specific cell type to the hypertrophic response by our comparison of wild type and ICAM-1-/- mice. Each of the cell types found to express ICAM-1 could contribute to the hypertrophic response to muscle overload via direct and/or indirect mechanisms.

A novel finding of the present study was the overload-induced expression of ICAM-1 by myofibers and satellite cells/myoblasts. Prior investigators demonstrated that human skeletal muscle cells in vitro [23], [24], [25], [26] and in vivo [27], [28] do not constitutively express ICAM-1. Accordingly, we did not detect ICAM-1 in our control cultures of proliferating, differentiating, or terminally differentiated skeletal muscle cells (C2C12 and primary cells), nor did we find ICAM-1 to be expressed by myofibers in control muscles. In contrast, myofiber expression of ICAM-1 has been observed in muscle biopsies obtained from patients with inflammatory myopathies [27], [28]. In these patients, ICAM-1 was expressed by necrotic [27], regenerating [27], [28], and normal myofibers [28]. In the present study, the ICAM-1+ myofibers were classified as normal myofibers as they did not display hallmark features of injury, necrosis, or regeneration. Our observations demonstrate that induced expression of ICAM-1 by myofibers can occur in non-pathological conditions and in the absence of overt muscle damage.

The function of ICAM-1 expressed by myofibers in overloaded muscles remains to be determined. The apparent clustering of ICAM-1 on the membrane of myofibers may be physiologically relevant as dimerization of ICAM-1 enhances binding affinity for β2 integrins and activates ICAM-1 signaling [22]. We speculate that the expression of ICAM-1 on the membrane of myofibers serves as a means by which myeloid cells can adhere to myofibers via a β2 integrin dependent mechanism. Adhesion-induced activation of β2 integrins and ICAM-1 could, in theory, serve as a unifying mechanism by which myeloid cells [18], β2 integrins [17], and ICAM-1 (present study) contribute to hypertrophy after muscle overload. Specifically, ligation-induced activation of β2 integrins and ICAM-1 initiates the release of cytokines and ROS from myeloid cells [19], [49] and activates signaling pathways (e.g., MAPK and PI3K/Akt) and cytokine production in ICAM-1 expressing cell types [20], [21], [29], [32]. Thus, myeloid cell adhesion to myofibers via a β2 integrin-ICAM-1 dependent mechanism could contribute to overload-induced hypertrophy via adhesion-induced release of cytokines from myeloid cells and/or activation of ICAM-1 signaling in myofibers. Central elements of our working model are consistent with our findings that ICAM-1-/- (present study) and CD18-/- [17] mice showed little to no signs of hypertrophy after muscle overload and that ICAM-1+ myofibers were found in overloaded muscles of CD18-/- mice (present study). We interpret these findings to indicate that expression of ICAM-1 on the membrane of myofibers after muscle overload per se is not sufficient to promote hypertrophy and that interactions between β2 integrins and ICAM-1 are necessary for overload-induced skeletal muscle hypertrophy.

The function of ICAM-1 by a population of satellite cells/myoblasts in overloaded muscles also remains to be determined. Compared to wild type mice, we found greater elevations in markers of satellite cell/myoblast proliferation at 3 d of overload and a lower percentage of regenerating myofibers at 7 d of overload in both ICAM-1-/- (present study) and CD18-/- mice [17]. As ICAM-1 and β2 integrins serve as a mechanism through which leukocytes cause death of transplanted myoblasts [50], [51], ICAM-1 expression by satellite cells/myoblasts could acutely reduce the number of myoblasts in overloaded muscles by serving as a means by which β2 integrin expressing leukocytes (e.g., neutrophils) adhere to and cause death of myoblasts. Alternatively, the expression of ICAM-1 by satellite cells/myoblasts in overloaded muscles could facilitate the formation of regenerating myofibers by enhancing myoblast-myoblast adhesion/fusion [23], [52], and/or serve as a means by which satellite cells/myoblasts adhere to and contribute nuclei to hypertrophying myofibers [53]. The extent to which satellite cells/myoblasts and regenerating myofibers contribute to overload-induced hypertrophy however, remains controversial [40], [54].

Endothelial cell expression of ICAM-1 could contribute to the hypertrophic response by influencing the number of myeloid cells that accumulate in overloaded muscles. We previously reported that neutrophil accumulation after muscle overload occurred via a β2 integrin independent (3 d of overload) and a β2 integrin dependent (7 d of overload) mechanism [17]. In the present study, neutrophil accumulation was found to occur via an ICAM-1 independent (3 d of overload) and an ICAM-1 dependent (7 d of overload) mechanism. As previously discussed [17], the substantial edema observed at 3 d of overload may have allowed neutrophils to bypass β2 integrin-ICAM-1 mediated firm adhesion and enter skeletal muscle by adhering to junctional adhesion molecules on endothelial cells, which is the final adhesion event of neutrophil diapedesis [55]. Macrophage accumulation after muscle overload on the other hand, was found to be independent of β2 integrins [17] and ICAM-1 (present study). These findings are in agreement with prior work which reported that adhesion and transendothelial cell migration of monocytes can occur via a β1 integrin (VLA-4) and VCAM-1 dependent mechanism [56], [57].

Little is known on the contribution of myeloid cells to skeletal muscle hypertrophy. Specifically, no investigation to our knowledge has examined the contribution of neutrophils to the hypertrophic response to muscle overload; whereas, DiPasquale et al. [18] reported that reductions in macrophages via clodronate liposome treatment reduced hypertrophy after muscle overload. In the present study, the impaired hypertrophy in overloaded ICAM-1-/- mice was associated with reductions in neutrophils, but not macrophages. We do not interpret these findings to indicate that neutrophils, but not macrophages, contribute to hypertrophy after muscle overload for several reasons. One, no evidence exists on the contribution of neutrophils to hypertrophy after muscle overload. Two, even if neutrophils influence the hypertrophic response, the impaired hypertrophy in overloaded ICAM-1-/- mice could also be attributable to a reduced ability of macrophages to adhere to skeletal muscle cells and/or to impairments in their ability to produce cytokines [19], [20]. Thus, the myeloid cell accumulation profile observed in the present study provides no insight into the specific contribution of neutrophils and/or macrophages to hypertrophy after muscle overload.

The mechanism(s) through which mechanical loading of skeletal muscle induces ICAM-1 expression by myofibers and satellite cells/myoblasts remains to be determined. Several of the cytokines that induce ICAM-1 expression by cultured myoblasts or myotubes (e.g., TNF-α, IL-1β, IL-6, and IL-4) [23], [24], [25], [26] have been reported to be elevated at the gene and/or protein level in skeletal muscle after mechanical loading [58], [59], [60]. In addition to cytokines, in vitro mechanical strain [61] and fluid shear stress [62] have been reported to increase endothelial cell expression of ICAM-1. Thus, increased expression of ICAM-1 after muscle overload may have been the result of mechanical loading-induced cytokine production and/or through a mechanosignal transduction mechanism.

Our current findings extend prior work on the immunobiology of skeletal muscle hypertrophy by demonstrating that myofibers and satellite cells/myoblasts express ICAM-1 after muscle overload, and that ICAM-1 contributes to the ensuing hypertrophic response. Because ICAM-1 is a major ligand for the β2 integrin CD11b expressed by myeloid cells, we propose that the expression of ICAM-1 by myofibers after muscle overload serves as a means by which myeloid cells bind to and initiate cell-to-cell communication with them. β2 integrin-ICAM-1 interactions could augment the hypertrophic response to muscle overload via adhesion-induced release of cytokines from myeloid cells and/or via activation of ICAM-1 signaling in myofibers. Further work is needed to elucidate the specific contribution of ICAM-1 expressed by skeletal muscle cells to overload-induced hypertrophy, and the influence of β2 integrin-ICAM-1 interactions on the phenotype of skeletal muscle cells. Understanding mechanisms by which the inflammatory response regulates growth processes in skeletal muscle could lead to the development of new approaches for rehabilitating musculoskeletal injuries, as well as for promoting the maintenance and/or growth of skeletal muscle, particularly in older individuals and those with inflammatory muscle, cardiovascular, and/or metabolic disease.

## Supporting Information

Figure S1 **ICAM-1 localization in differentiated cultures of C2C12 and primary cells after TNF-α treatment.** Cells were treated with TNF-α (10 ng/ml) for 24 h. Cells were fixed in 50% methanol/50% acetone, permeabilized with 0.2% triton-X100, and incubated for 2 h with an antibody that recognizes an extracellular domain of mouse ICAM-1 (1:50; R&D Systems product # AF796). Detection of ICAM-1 (green) was achieved using an Alexa Fluor® 488-conjugated secondary antibody and nuclei were stained with DAPI (blue). Treatment of differentiated cultures with TNF-α resulted in the expression of ICAM-1 by multinucleated myotubes and in non-fused myoblasts in both C2C12 and primary cells. ICAM-1 was not detected in control cultures (not shown).
(TIF)

Figure S2 **Immunohistochemistry detection of ICAM-1 in plantaris muscles of wild type mice.** In muscle sections of control muscles, ICAM-1 was found only in presumptive blood vessels (arrowheads). Muscle sections of 3, 7, and 14 d overloaded muscles showed ICAM-1 on the membrane of myofibers (arrows) and in cells residing in the interstitium.
(TIF)

Figure S3 **ICAM-1 localization in CD18-/- mice.** Confocal microscopy images of control (CT) and 7 and 14 d overloaded muscles. In control muscles, ICAM-1 (green) was found to be expressed by presumptive endothelial cells (DAPI; blue) neighboring myofibers. Muscles overloaded for 7 or 14 d showed ICAM-1 expression (green) on the membrane of myofibers (WGA; red) and by cells (DAPI; blue) residing in the interstitium. Column labeled as “MERGED” include ICAM-1, WGA, and DAPI images.
(TIF)
