# Supplementary figures and images for: Skeletal Muscle Cells Express ICAM-1 after Muscle Overload and ICAM-1 Contributes to the Ensuing Hypertrophic Response
Source: PLoS One. 2013 Mar 11;8(3):e58486. doi: 10.1371/journal.pone.0058486 (PMC3594308; doi:10.1371/journal.pone.0058486)

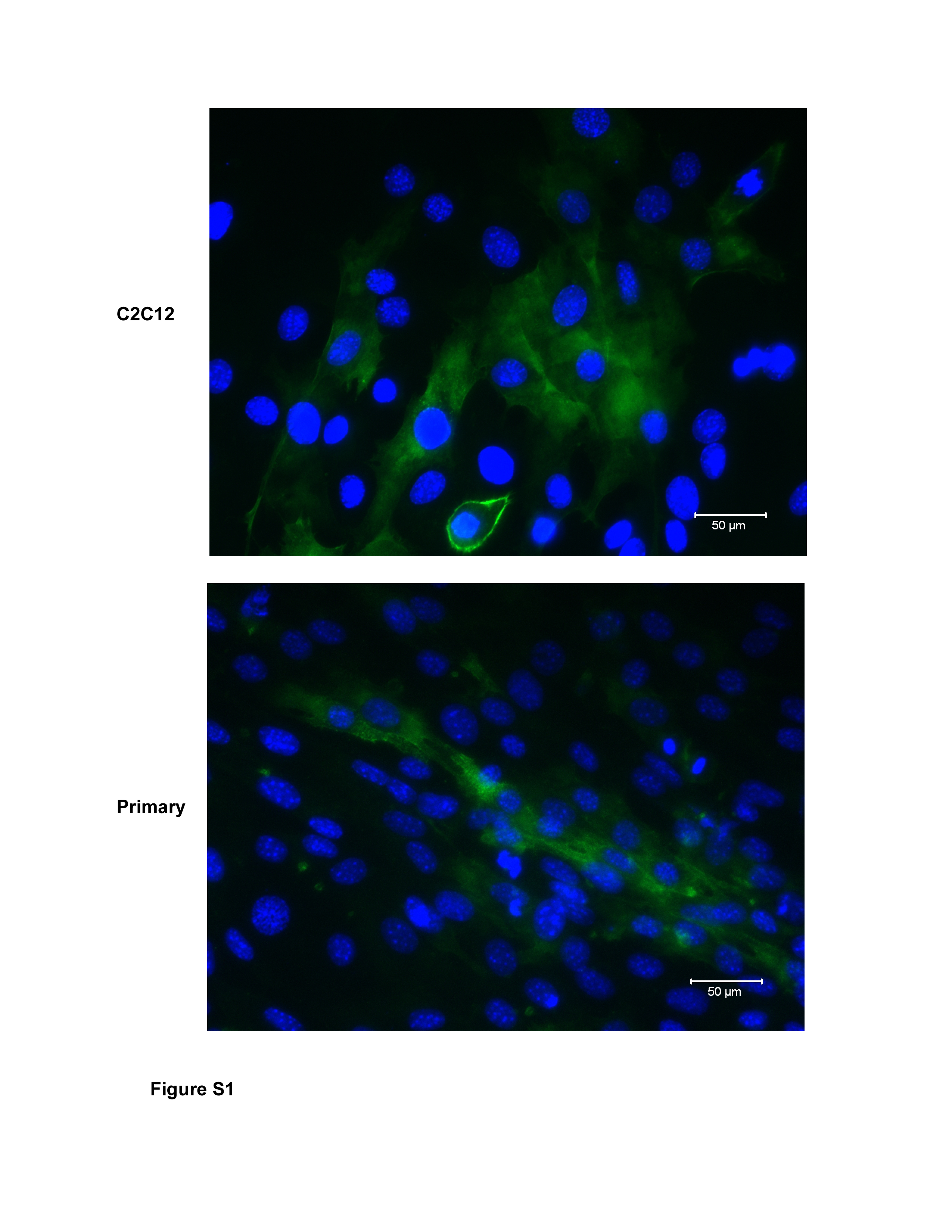

Supplement: Figure S1 — ICAM-1 localization in differentiated cultures of C2C12 and primary cells after TNF-α treatment. Cells were treated with TNF-α (10 ng/ml) for 24 h. Cells were fixed in 50% methanol/50% acetone, permeabilized with 0.2% triton-X100, and incubated for 2 h with an antibody that recognizes an extracellular domain of mouse ICAM-1 (1:50; R&D Systems product # AF796). Detection of ICAM-1 (green) was achieved using an Alexa Fluor® 488-conjugated secondary antibody and nuclei were stained with DAPI (blue). Treatment of differentiated cultures with TNF-α resulted in the expression of ICAM-1 by multinucleated myotubes and in non-fused myoblasts in both C2C12 and primary cells. ICAM-1 was not detected in control cultures (not shown). (TIF) [file pone.0058486.s001.tif]

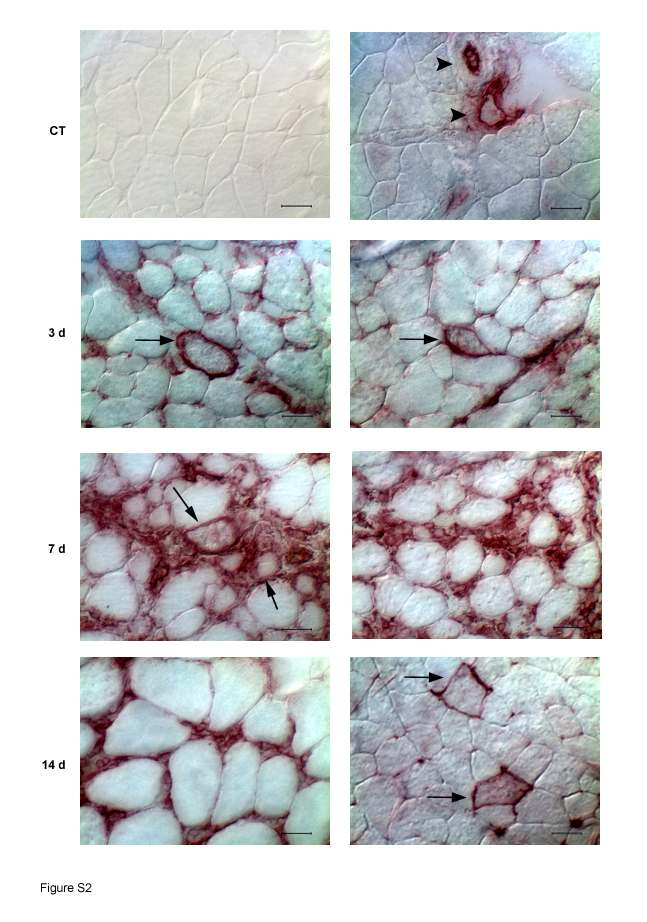

Supplement: Figure S2 — Immunohistochemistry detection of ICAM-1 in plantaris muscles of wild type mice. In muscle sections of control muscles, ICAM-1 was found only in presumptive blood vessels (arrowheads). Muscle sections of 3, 7, and 14 d overloaded muscles showed ICAM-1 on the membrane of myofibers (arrows) and in cells residing in the interstitium. (TIF) [file pone.0058486.s002.tif]

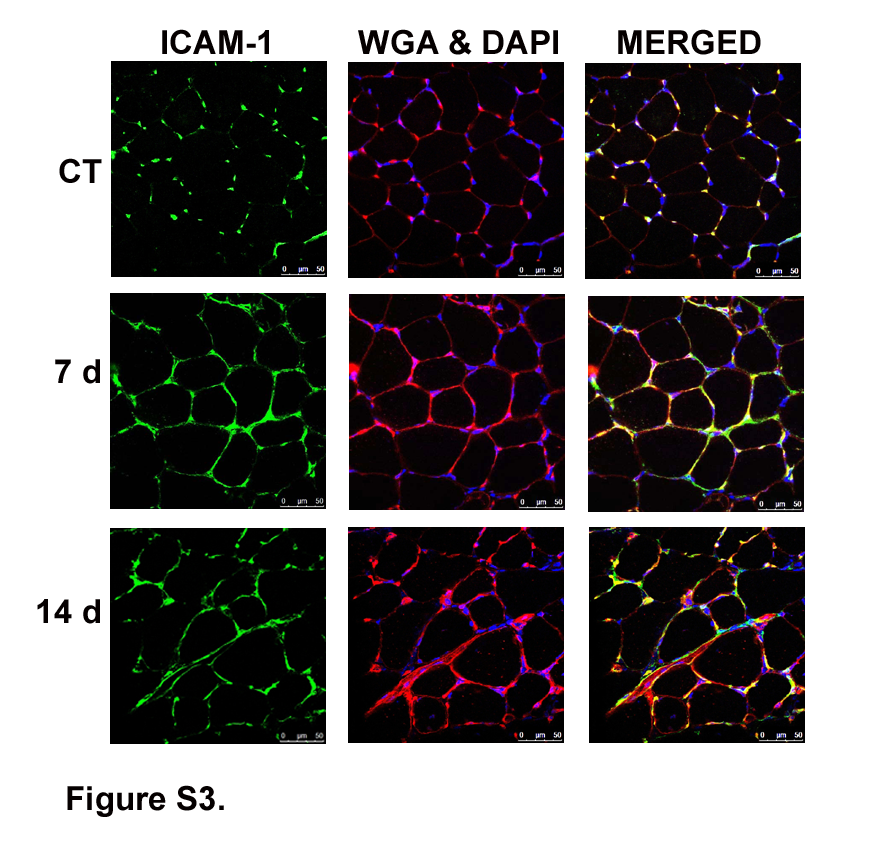

Supplement: Figure S3 — ICAM-1 localization in CD18-/- mice. Confocal microscopy images of control (CT) and 7 and 14 d overloaded muscles. In control muscles, ICAM-1 (green) was found to be expressed by presumptive endothelial cells (DAPI; blue) neighboring myofibers. Muscles overloaded for 7 or 14 d showed ICAM-1 expression (green) on the membrane of myofibers (WGA; red) and by cells (DAPI; blue) residing in the interstitium. Column labeled as “MERGED” include ICAM-1, WGA, and DAPI images. (TIF) [file pone.0058486.s003.tif]
